# Integrated transcriptome and metabolome analyses reveal the adaptation of Antarctic moss *Pohlia nutans* to drought stress

**DOI:** 10.3389/fpls.2022.924162

**Published:** 2022-08-11

**Authors:** Shuo Fang, Tingting Li, Pengying Zhang, Chenlin Liu, Bailin Cong, Shenghao Liu

**Affiliations:** ^1^Key Laboratory of Marine Eco-Environmental Science and Technology, First Institute of Oceanography, Ministry of Natural Resources, Qingdao, China; ^2^National Glycoengineering Research Center, School of Life Sciences, Shandong University, Qingdao, China; ^3^School of Advanced Manufacturing, Fuzhou University, Jinjiang, China

**Keywords:** abiotic stress, flavonoids, metabolome, transcriptome, abscisic acid, bryophytes

## Abstract

Most regions of the Antarctic continent are experiencing increased dryness due to global climate change. Mosses and lichens are the dominant vegetation of the ice-free areas of Antarctica. However, the molecular mechanisms of these Antarctic plants adapting to drought stress are less documented. Here, transcriptome and metabolome analyses were employed to reveal the responses of an Antarctic moss (*Pohlia nutans* subsp. LIU) to drought stress. We found that drought stress made the gametophytes turn yellow and curled, and enhanced the contents of malondialdehyde and proline, and the activities of antioxidant enzymes. Totally, 2,451 differentially expressed genes (DEGs) were uncovered under drought treatment. The representative DEGs are mainly involved in ROS-scavenging and detoxification, flavonoid metabolism pathway, plant hormone signaling pathway, lipids metabolism pathway, transcription factors and signal-related genes. Meanwhile, a total of 354 differentially changed metabolites (DCMs) were detected in the metabolome analysis. Flavonoids and lipids were the most abundant metabolites and they accounted for 41.53% of the significantly changed metabolites. In addition, integrated transcriptome and metabolome analyses revealed co-expression patterns of flavonoid and long-chain fatty acid biosynthesis genes and their metabolites. Finally, qPCR analysis demonstrated that the expression levels of stress-related genes were significantly increased. These genes included those involved in ABA signaling pathway (*NCED3*, *PP2C*, *PYL*, and *SnAK2*), jasmonate signaling pathway (*AOC*, *AOS*, *JAZ*, and *OPR*), flavonoid pathway (*CHS*, *F3’,5’H*, *F3H*, *FLS*, *FNS*, and *UFGT*), antioxidant and detoxifying functions (*POD*, *GSH-Px*, *Prx* and *DTX*), and transcription factors (*ERF* and *DREB*). In summary, we speculated that *P. nutans* were highly dependent on ABA and jasmonate signaling pathways, ROS scavenging, flavonoids and fatty acid metabolism in response to drought stress. These findings present an important knowledge for assessing the impact of coastal climate change on Antarctic basal plants.

## Introduction

The Southern Ocean and Antarctic coastal seas are undergoing large-scale changes in environmental conditions as a result of warming, ozone depletion, and a positive Southern Annular Mode (Robinson et al., 2018; Rogers et al., 2020; Cannone et al., 2022). In recent decades, global warming has led to a rapid warming of surface air temperatures on the Antarctic Peninsula, causing the accelerated melting of Antarctic glaciers (Lee et al., 2017; Hsu et al., 2021). In contrast to the most rapid warming of the Antarctic Peninsula, most regions of East Antarctica are experiencing drying climate and colder summer (Malenovský et al., 2015; Robinson et al., 2018). The more positive Southern Annular Mode driven by ozone depletion and greenhouse gas emissions is freezing the temperatures in East Antarctica in recent decades (Robinson et al., 2018). Furthermore, the Madden-Julian oscillation convection has become more active in the equatorial western Pacific, but gradually decreased in the tropical Indian Ocean, which provides driving forces for cooling East Antarctica (Hsu et al., 2021). The freezing temperatures subsequently cause the drying climate in this region. The decreased moss abundance, declining health of moss-beds, and long-term observations of lake salinity and climate on Windmill Island have together indicated the drying trend across the East Antarctica (Robinson et al., 2018).

The Antarctic terrestrial and maritime ecosystem suffers from a variety of harsh climatic conditions like extreme dryness, low temperature, high UV-B radiation, extremely short growing season, all of which severely limit the growth and development of plants (Convey and Peck, 2019; Perera-Castro et al., 2021; Liu et al., 2021b). Lichens and bryophytes as well as two native Antarctic vascular plants *Deschampsia antarctica* and *Colobanthus quitensis* dominate the vegetations of Antarctica Peninsula (Robinson et al., 2010). The maritime Antarctica, like the Antarctic Peninsula, has experienced extensively environmental changes and global warming, which has further led to accelerated expansion of *D. antarctica* and *C. quitensis* in 2009–2018 (Cavieres et al., 2016; Cannone et al., 2022). The native vascular plant *C. quitensis* in the Antarctic Peninsula likely relies on leaf CO_2_ transfer to promote leaf carbon uptake and growth in response to climate change (Sáez et al., 2018). Using an open top chamber with an increase of about 4°C at midday to mimick the effect of global warming, a detailed examination of the upregulated and downregulated proteins revealed substantial metabolic reprogramming, resulting in improved photoprotection and oxidative stress control, as well as lower cell wall component composition (Bertini et al., 2021). Additionally, fungi endophytes may contribute to the adaptation of *C. quitensis* to the extreme cold and aridity of Antarctica (Hereme et al., 2020). The Antarctic moss *Sanionia uncinata*, collected from King George Island, harbours desiccation-tolerant properties which are confirmed by analyses of physiological, antioxidant, and biochemical parameters (Pizarro et al., 2019). In Antarctic terrestrial systems, vegetation distributions are mainly determined by water availability of ice-free habitats, rather than temperature (Chown et al., 2015). Small changes in the microclimate can influence the freeze–thaw cycles or water availability and thus impact species distributions. For instance, the well-developed moss communities in Windmill Islands, East Antarctica, are now changing rapidly and the health of moss-beds is declining in response to regional dryness (Malenovský et al., 2015). These plants often struggle with sub-zero temperatures, strong winds and extreme dryness (Perera-Castro et al., 2020). Consequently, these environmental stressors will damage cell structure, cause oxidative stress, and destroy the physiological functions of Antarctic terrestrial and maritime plants (Cho et al., 2018).

Drought is one of the most unfavorable environmental stressors with different frequency, severity and duration (Zhang et al., 2014). Owing to climate change, the frequency and duration of drought periods will increase, and the impact of drought has become one of the most important threats (Trenberth et al., 2013; Zhao and Dai, 2015). Drought stress can cause photoinhibition and photodamage of photosynthetic apparatus, resulting in inhibition of plant photosynthesis, growth retardation and yield reduction (Munné Bosch et al., 2004). Meanwhile, the imbalance between the generation of reactive oxygen species (ROS) and their removal causes oxidative stress under drought stress (Cruz de Carvalho, 2008). Extra reactive oxygen species are very hazardous and can damage nucleic acids, proteins, and lipids due to their high reactivity (Osmolovskaya et al., 2018). To deal with environmental stress, plants have developed anti-stress strategies that include intracellular physiological and metabolic changes such as regulating cell membrane signal transduction, increasing cell membrane fluidity, and raising the synthesis of ROS-scavenging genes (Xiong et al., 2002). Membranes are the primary targets of drought-induced degradation processes, and the reduced membrane lipid content is associated with the stimulation of lipid activity and peroxidation activity under water stress (Gigon et al., 2004). Very long chain fatty acids (VLCFAs) are specifically present in several membrane lipids and essential for membrane homeostasis under the influence of abiotic stresses (Batsale et al., 2021). Notably, flavonoids are secondary metabolites widely distributed in terrestrial plants. They can relieve the damage of ROS to plants (Liu et al., 2013; Ma et al., 2014; Wang et al., 2020). Flavonoids and anthocyanins have strong free radical scavenging activities, which can help alleviate oxidative and drought stress in Arabidopsis (Nakabayashi et al., 2014; Wang et al., 2020). Furthermore, the abscisic acid (ABA) signaling system, which is involved in regulating stomatal conductance, generating antioxidant enzymes, accumulating osmotic agents, and producing LEA protein, regulates several plant responses to dryness (Raghavendra et al., 2010; Ali et al., 2020; Nawaz and Wang, 2020). The moss *Physcomitrella patens* and *Arabidopsis* have a conserved ABA signaling pathway where ABA binding to ABA receptors (RCARs/PYR1/PYLs) results in inactivation of type 2C protein phosphatases (PP2Cs; Raghavendra et al., 2010). Since bryophytes lack the appropriate rhizome, conduction vascular tissue, and porous epidermal pores to limit water loss, they rely mostly on inherent biological systems to counteract the damage induced by drought stress (Charron and Quatrano, 2009).

Bryophytes and lichens are the dominant organisms on the Antarctic continent (Pizarro et al., 2019). Bryophytes have an evolutionary trait that allows them to settle in low-water locations more easily than other species (Deane-Coe and Stanton, 2017). *Pohlia nutans* is one of Antarctica’s most common mosses. *P. nutans* can survive and propagate under harsh conditions. We investigated the global gene expression profiles under drought stress using transcriptome sequencing and identified 2,451 differentially expressed genes (DEGs). Meanwhile, we employed the UPLC–MS/MS platform to make a high-throughput analysis of the metabolites of *P. nutans* and detected 354 differentially changed metabolites (DCMs). Among them, flavonoids and fatty acids were the most significantly changed metabolites. An integrated analysis demonstrated that the accumulation of flavonoids was accompanied by the upregulation of flavonoid synthase genes, indicating flavonoid biosynthesis pathway might play an important role in the adaptation of *P. nutans* to drought stress.

## Experimental procedures

### Plant sample collection and drought stress treatment

The *P. nutans* subsp. LIU was collected near the Great Wall Station on the Fildes Peninsula (S62°13.260′, W58°57.291′) in Antarctica in March 2014. Plants were grown on a mixed soil (Krasman-Delman, Germany) and cultured at 16°C, 50 μmol photons·m^−2^·s^−1^ light with a 16-h-light/8-h-dark photoperiod (Liu et al., 2017). To retain moisture, the gametophytes were covered with plastic film. The plants were sprayed with PEG6000 solution (20%) to simulate drought stress. When plants are subjected to abiotic stresses, they first perceive stress signals and initiate the gene expression of signaling pathways, followed by synthesizing the associated enzymes, and then generate metabolites (Zhang et al., 2022). Plants respond to stress in a logical order in terms of gene expression, physiological responses, and biochemical reactions. In the present study, the transcriptome sequencing of *P. nutans* were conducted at 0 and 2 h after PEG6000 treatment to identify genes involved in sensing and signal transduction. To confirm transcriptome sequencing results, we performed quantitative real-time RT-PCR analysis of plants treated with PEG6000 for 0, 2, 12, and 24 h. Since the accumulation of metabolites was delayed compared with the time of gene expression, we used the LC–MS/MS method to identify metabolites in gametophytes at 0, 24, and 60 h after PEG6000 treatment (i.e., CG, PEG24h, and PEG60h). The plants without PEG6000 treatment were collected as the control group (CG). The samples were quickly frozen in liquid nitrogen and stored at −80°C.

### Biochemical features of *Pohlia nutans* under drought stress

In order to be consistent with the treatment times of LC–MS/MS analysis, plants were treated with PEG6000 by 0 h, 12 h, 24 h, 60 h for biochemical experiments. We employed commercial kits that were all purchased from Nanjing Jiancheng Bioengineering Institute (Nanjing, China) to detect biochemical characteristics. Briefly, 1 g of moss gametophytes with various treatment times were cut and ground into powders in liquid nitrogen. Then, 0.1 g of the powder was added to the appropriate extracting buffer to generate a 10% tissue homogenate. The supernatant was obtained after centrifugation at 4000 r/min for 10 min. The supernatant was used to detect the contents of malondialdehyde (MDA) and proline (PRO) using the commercial kits (Product No. A003-2-3 and A107-1-1), respectively. Meanwhile, to measure the enzyme activities of peroxidase (POD), catalase (CAT), ascorbate peroxidase (APX), superoxide dismutase (SOD) and glutathione peroxidase (GSH-PX), the samples were homogenized in physiological saline to make 10% tissue solution and the supernatant were collected after centrifugation at 4000 × *g*/min for 10 min. POD catalyzed the reaction of hydrogen peroxide (H_2_O_2_) and the enzyme activity was calculated by measuring the absorbance values at 420 nm (Product No. A084-3-1). CAT decomposing H_2_O_2_ could be quickly terminated by adding ammonium molybdate, and the remaining H_2_O_2_ reacted with ammonium molybdate producing a pale-yellow complex. The absorbance values were measured at 405 nm, and the activity of CAT was calculated (Product No. A007-1-1). APX was mainly present in chloroplasts, and its activity was determined by catalyzing the reaction of ascorbic acid with H_2_O_2_ (Product No. A123-1-1). GSH-PX could promote the reaction of H_2_O_2_ and reduce glutathione to generate water and oxidized glutathione. The activity of GSH-PX could be expressed by the speed of its enzymatic reaction (Product No. A005-1). SOD activity was based on a plate assay using xanthine oxidase and the water-soluble tetrazolium (WST-1 dye; Peskin and Winterbourn, 2017). The SOD activity was measured at 450 nm by using superoxide dismutase assay kit (Product No. A001-3). All of the above experiments were performed in three biological replicates and three technical replicates.

### RNA extraction and transcriptome sequencing

Trizol reagent (Thermo Fisher Scientific, Waltham, MA, United States) was used to extract total RNA from the samples, and the RNA quality was analyzed by 1% agarose gel electrophoresis. The integrity of RNA was tested using Agilent RNA Nano6000 kit and Agilent 2,100 bioanalyzer (Agilent Technologies, California, United States). The concentration of total RNA was measured using Qubit® RNA Assay Kit (Thermo Fisher Scientific). Magnetic beads were used to purify mRNA and then to break it down into smaller fragments. Following the manufacturer’s instructions, sequencing libraries were generated using the NEBNext®UltraTM RNA Library Prep Kit for Illumina® (New England Biolabs, Ipswich, United States). Finally, the libraries were sequenced on Illumina Hiseq 2500 platform by Novogene Biotech Co., Ltd. (Beijing, China).

In order to improve the data quality, we deleted the sequencing adapters or poly-N from the raw data, where N represented the original read with uncertain base sequence. The low-quality reads (bases with Qphred ≤5 accounted for more than 50% of the sequence) were also removed. The clean reads were generated and used for the following analysis. Firstly, we performed the alignment program for mapping sequencing reads to the reference genome of *P. nutans* (Liu et al., 2022). This process was conducted by HISAT2 program, generating a bam format file with index information (Kim et al., 2019). We then used featureCounts program to assign sequence reads to genes and acquire the read summarization (Liao et al., 2014). We employed the Transcripts Per Kilobase of exon model per Million mapped reads (TPM) method to calculate the expression value of each transcript. Finally, significance of gene expression levels between PEG6000-treatment and Control was assessed using the DESeq R package. Differentially expressed genes (DEGs) was identified by using the threshold of value of *p* < 0.05 and |log_2_(Fold Change)| > 1. DEGs were annotated using Swiss-Prot databases. GO enrichment analysis of the DEGs was implemented by the TBtools software (Chen et al., 2020). KEGG pathway enrichment was performed using clusterProfiler (Wu et al., 2021).

### Quantitative real-time RT-PCR analysis (qPCR)

We employed the qPCR technique to evaluate the precision of gene expression analysis in transcriptome sequencing. Plant samples were ground into powder with liquid nitrogen. RNA was extracted using a *TransZol* Up Plus RNA Kit (TransGen, Beijing, China). 500 ng of total RNA were employed for cDNAs synthesis using *TransScript*® All-in-One First-Strand cDNA Synthesis Super Mix Kit (TransGen). We tested four house-keeping genes (*GAPDH*, tubulin beta-7, tubulin beta-1, and actin 1) and finally chose *GAPDH* as reference gene, because it was stable expression under control condition and drought stress (Li et al., 2021). We designed gene specific primers for qPCR analysis to detect the expression levels of genes involved in flavonoid synthesis, ROS-scavenging, abscisic acid (ABA) and jasmonic acid (JA) pathways. The gene specific primers were listed in Supplementary Table 1. The qPCR experiments were performed on the Light Cycler® 96 Instrument (Roche, Basel, Switzerland) using *Perfect Start*® Green qPCR Super Mix Kit (TransGen). The cycling regime was 95°C for 2 min, followed by 45 amplification cycles (94°C for 5 s, 60°C for 15 s, and 72°C 10 s). The reaction was repeated three times for each template. The experiment was repeated three times. Relative gene expression levels were calculated by using the Ct(2^−ΔΔCt^) method (Livak and Schmittgen, 2001).

### Metabolite extraction and LC-MS/MS analysis

Plant samples were freeze-dried using a vacuum freeze dryer (Scientz, Ningbo, China). The samples were then ground with a hybrid grinder (MM400, Retsch, Germany). To dissolve lyophilized powder, 1.2 ml of 70% aqueous methanol was added to the plant samples at 4°C overnight. The supernatant was obtained after centrifugation at 10,000 × *g* for 10 min, and filtered using a 0.22 μm membrane (ANPEL, Shanghai, China). The supernatant was subjected for LC–MS/MS analysis and conducted by Wuhan Metware Biotechnology Co., Ltd. following the standard procedure (Jang et al., 2018; Wang et al., 2021). We used the ultra-performance liquid chromatography (Shim-pack UFLC CBM30A, Shimadzu, Japan) and tandem mass spectrometry (Applied biosystems, Framingham, United States) for metabolome analysis. Metabolites from all samples were subjected to orthographic projection of primary component analysis (PCA) and potential structure identification analysis (OPLS-DA). Metabolites with fold change ≥2 or fold change ≤0.5 and variable importance in project (VIP) ≥1, were defined as differentially changed metabolites (DCMs). Enrichment analysis of DCMs was performed using the KEGG database.

### Statistical analysis

Data are expressed as mean ± standard error of the mean (SEM). Significant differences between the drought group and CG were calculated using Student’s *t*-test (*p < 0.05, **p < 0.01).

## Results

### Biochemical parameters and phenotypic changes under drought stress

**Figure 1:**
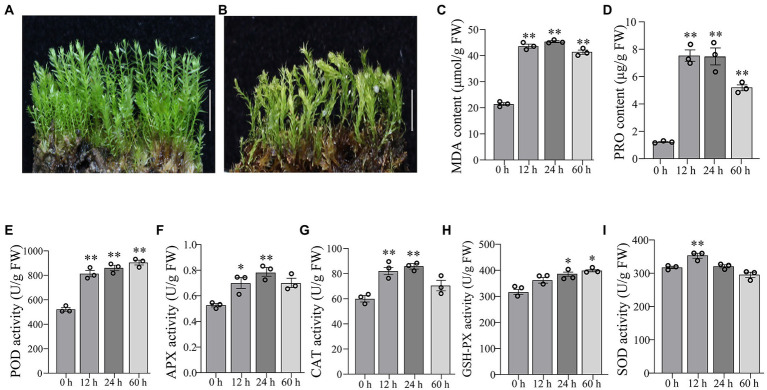
The moss *Pohlia nutans* can tolerance strong drought stress. **(A)** Photo of *P. nutans* cultured under normal condition. **(B)** Photo of *P. nutans* treated with PEG6000 for 60 h to simulate the drought stress. **(C–I)** The biochemical features of *P. nutans* under PEG6000 treatment. All experiments were repeated three times. Bar = 1.0 cm. Significant difference (*p < 0.05, **p < 0.01).

The gametophytes of *P. nutans* were shown in Figure 1A. The gametophytes turned yellow after 60 h of 20% PEG6000 solution treatment and the stems curled and dried out (Figure 1B). We conducted biochemical experiments on PEG6000-treatment and CG. MDA content was significantly increased at 12 and 24 h after PEG6000 treatment (Figure 1C). PRO content was significantly increased at 12 and 24 h of PEG6000 treatment (Figure 1D). POD activity increased 1.55-fold, APX activity increased 1.38-fold, and CAT activity increased 1.45-fold in *P. nutans* after PEG6000 treatment for 12 h (Figures 1E–G). When the *P. nutans* treated with PEG6000 for 24 h, POD activity increased by 1.65-fold, CAT increased by 1.43-fold, APX increased by 1.48-fold, and GSH-PX activity increased by 1.34-fold (Figures 1E–H). The enzyme activity of SOD was slightly increased after PEG6000 treatment (Figure 1I). Our results showed that MDA accumulation occurred in plants under drought stress, indicating the generation of membrane lipid peroxidation. Meanwhile, the increased content of proline and enhanced antioxidant enzyme activities might contribute to improve the resistance of plants to drought stress.

### Transcriptome sequencing and gene differential expression analysis

**Figure 2:**
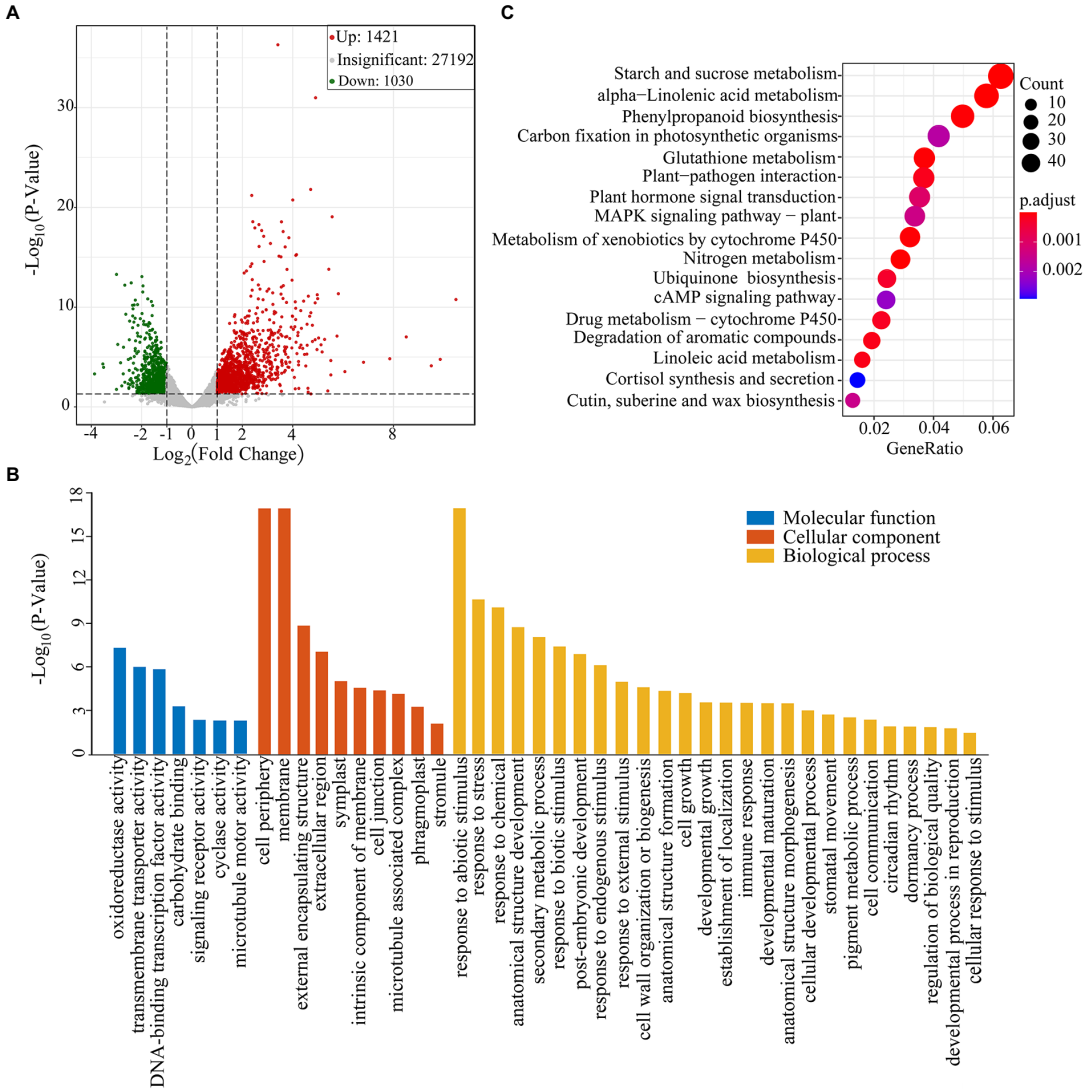
Transcriptome sequencing analysis of *Pohlia nutans* under drought stress. **(A)** Volcano plot of differentially expressed genes (DEGs) in CG and PEG-treated groups. Each point in the figure represents a gene. Green dots represent downregulated genes, red dots represent upregulated genes, and gray dots represent genes detected but not significantly different. **(B)** GO enrichment analysis of DEGs. **(C)** KEGG pathway enrichment of DEGs. Gene ratio represents the ratio of the number of DEGs to the total number of annotated genes in this pathway.

The TPM method was used to calculate gene expression levels. The obtained matrix was subjected to differential expression analysis, and the screening conditions were that the absolute value of log_2_(Fold change) was greater than 1.0, and the value of *p* was less than 0.05. A total of 2,451 DEG were identified between drought group and CG (Supplementary Table 2). Of them, 1,421 DEGs were upregulated and 1,030 DEGs were down-regulated (Figure 2A). We found that these DEGs were involved in ROS-scavenging and detoxification, flavonoid pathways and lipid metabolism. The DEGs were also involved in plant hormone metabolism pathway, including abscisic acid (ABA), jasmonic acid (JA), auxin and gibberellin pathways (Supplementary Table 3).

GO enrichment analysis showed that DEGs were divided into three functional groups: biological processes, cellular components, and molecular functions (Figure 2B). In the category of molecular function, we discovered that DEGs were predominant in classes associated with “oxidoreductase activity” and “transmembrane transporter activity.” In the category of biological process, DEGs were distributed into the GO classes including “response to abiotic stimulus,” “response to stress,” “anatomical structure development,” “response to chemical,” and “secondary metabolic process.” Subsequently, we conducted the KEGG enrichment analysis of DEGs. Several enriched pathways were involved in abiotic stresses, such as alpha-linolenic acid metabolism, phenylpropanoid biosynthesis and plant hormone signal transduction, as well as cutin, suberin and wax biosynthesis. Besides, several other pathways were also significantly enriched, including starch and sucrose metabolism, glutathione metabolism, and carbon fixation in photosynthetic organisms (Figure 2C).

**Figure 3:**
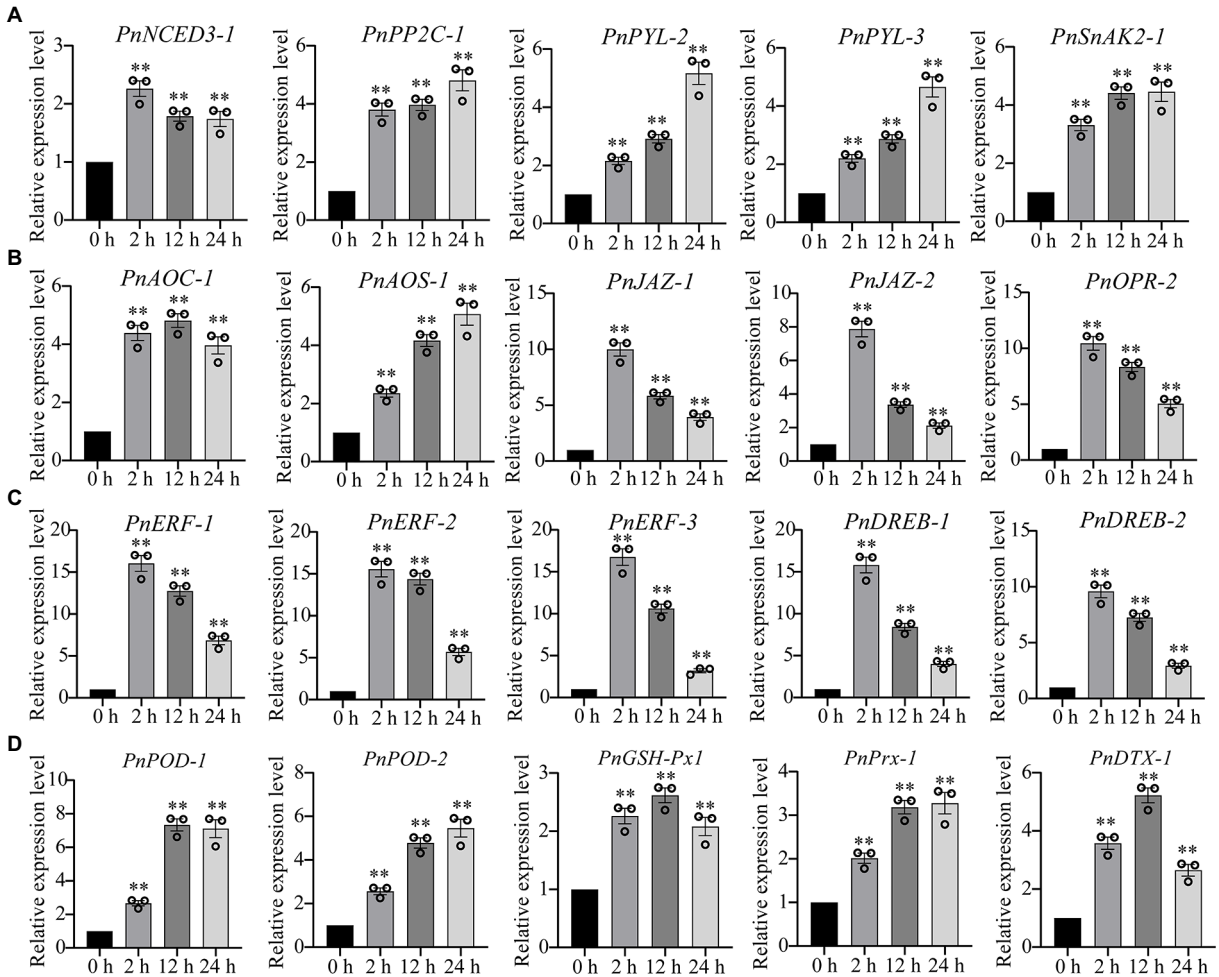
qPCR analysis confirmed the upregulation of several representative DEGs under drought stress. **(A)** The expression levels of genes involved in abscisic acid (ABA) signaling pathway. **(B)** The expression levels of genes involved in jasmonate signaling pathway. **(C)** The gene expression levels of ERF and DREB transcription factors. **(D)** The gene expression levels of antioxidant enzymes. NCED3, 9-cis-epoxycarotenoid dioxygenase; PYL, promoter-pyrabactin resistance; PP2C, type 2C protein phosphatases; SnAK2, SnRK1-activating protein kinase 2; OPR, 12-oxophytodienoate reductase; AOC, allene oxide cyclase; AOS, allene oxide synthase; JAZ, jasmonate ZIM domain protein; ERF, ethylene-responsive element binding factor; DREB, dehydration responsive element-binding transcription factor; POD, peroxidase; GSH-Px, glutathione peroxidase; Prx, peroxiredoxin; DTX, protein DETOXIFICATION. The data were calculated from three biological replicates. Vertical bars are means ±SE. Significant difference (**p < 0.01).

Our transcriptome sequencing showed that several genes involved in ABA biosynthesis and signaling pathway were upregulated, including ABA synthase (9-cis-epoxycarotenoid dioxygenase, NCED3), ABA receptor (promoter-pyrabactin resistance, PYL) and type 2C protein phosphatases (PP2C), as well as SnRK1-activating protein kinase 2 (SnAK2; Supplementary Table 3). Similarly, the several genes involved in jasmonate biosynthesis and signaling pathway were also significantly upregulated under drought stress, such as 12-oxophytodienoate reductase (*OPR*), allene oxide cyclase (*AOC*), allene oxide synthase (*AOS*), jasmonate ZIM domain protein (*JAZ*; Supplementary Table 3). We performed the qPCR analysis to verify the gene expression levels in ABA and JA signaling pathways. We found that the genes involved in ABA signaling pathway (*PnNCED3-1*, *PnPP2C-1*, *PnPRL-2*, *PnPYL-3*, *PnSnAK2-1*) and jasmonate signaling pathway (*PnAOC-1*, *PnAOS-1*, *PnJAZ-1*, *PnJAZ-2*, *PnOPR-2*) were markedly upregulated under drought stress (Figures 3A,B). Ethylene-responsive element binding factors (ERF) and dehydration responsive element-binding transcription factors (DREB) are two major subfamilies of the AP2/REF and play essential roles in the regulation of abiotic-stress responses, respectively. Our results demonstrated that *ERF* and *DREB* were markedly upregulated under drought stress (Figure 3C). In addition, the gene expression levels of several antioxidant enzymes were also significantly upregulated, including peroxidase (POD) and glutathione peroxidase (GSH-Px), peroxiredoxin (Prx), and protein DETOXIFICATION (DTX) that were responsible for ROS-scavenging in *P. nutans* under drought stress (Figure 3D).

### Metabolome analysis of *Pohlia nutans* to drought stress

**Figure 4:**
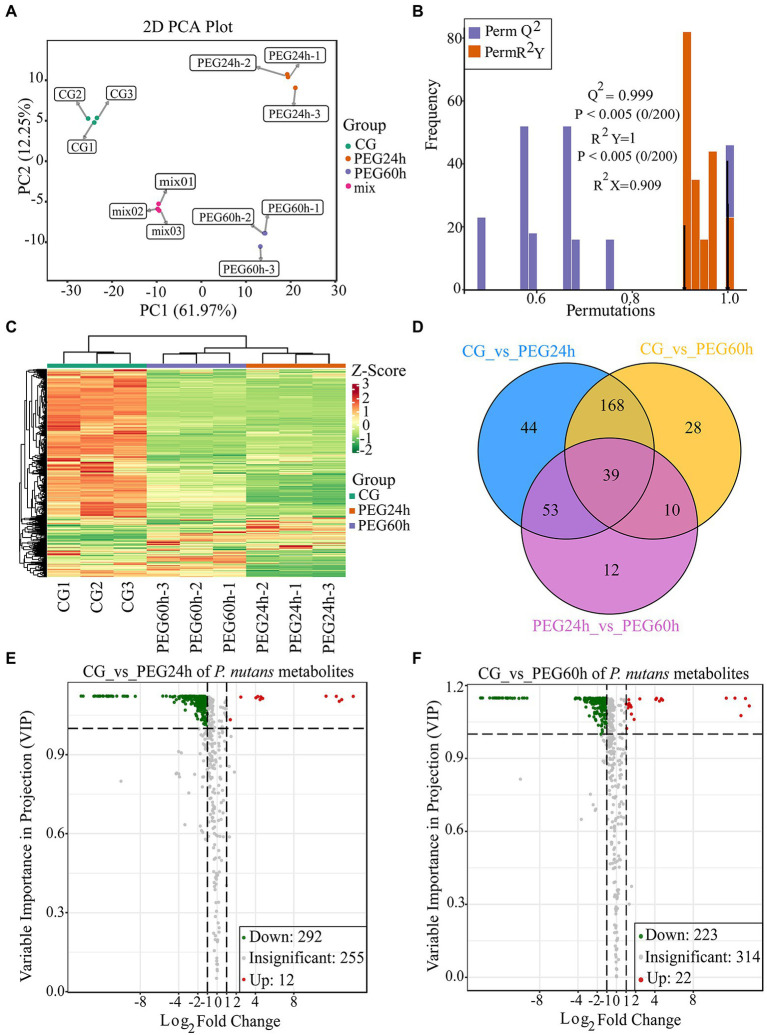
Widely targeted metabolome analysis of *Pohlia nutans* under drought stress. **(A)** Principal component analysis (PCA) for mass spectrum data under different groups. CG is control without PEG6000 treatment. Mix means the mixed samples. PEG is abbreviation of PEG6000. Here each group was replicated for three biologicals. **(B)** Orthogonal projections to latent structures discriminant analysis (OPLS-DA) for mass spectrum data. **(C)** Differentially changed metabolites (DCMs) clustering heatmap. Horizontal ordinate shows the sample name, and vertical ordinate is the DCMs. After hierarchical clustering of DCMs, the dendritic plot to the left of the heat map represents the results of DCMs clustering. **(D)** The Venn diagram showing the shared and unique DCMs in three different groups. **(E)** Volcano plot showing the DCMs in the PEG24 h vs. CG. **(F)** Volcano plot showing the DCMs in the PEG60 h vs. CG. Each dot represents one metabolite. The horizontal ordinate indicates the fold change of the metabolites between the two groups, and the VIP value indicates significant differences in the statistical analysis.

To detect the metabolite changes of *P. nutans* under drought stress, a widely targeted metabolome analysis was performed based on the UPLC–MS/MS platform. A total of 559 metabolites were obtained (Supplementary Table 4). The PCA results showed that the mixed samples were grouped together, suggesting that the detection technique was stable and reproducible. Moreover, there were significant differences among CG, PEG24h and PEG60h, and they were well clustered within the group (Figure 4A). OPLS-DA could effectively eliminate influences irrelevant to the experiment by incorporating supervised pattern recognition into the multivariate statistical analysis. The model verification permutation test of OPLS-DA was calculated (Figure 4B). The abscissa of the permutation test represents the accuracy of the model, and the ordinate represents the frequency of the accuracy of 200 models in the permutations test. The arrow indicates where the accuracy of the OPLS-DA model is located, and *Q*^2^ indicates the predictive power of the model. R^2^X and R^2^Y represent the interpretation rate of the X and Y matrices of the built model. Theoretically, the closer the values of *R*^2^ and *Q*^2^ are to 1, the better the model is. Our results showed that *Q*^2^ was greater than 0.9 and *p* values was less than 0.05 based on this permutation test, indicating that the constructed model for data analysis was suitable. The DCMs were then further screened using log_2_(Fold change) and VIP, and heatmaps were drawn to show the overall changes of metabolites (Figure 4C). Venn diagrams illustrated the differences in DCMs between different groups. A total of 39 compounds were shared among three groups, including lipids (1-oleoyl-sn-glycerol, 1-linoleoylglycerol-3-O-glucoside, ginger glycolipid B) and flavonoids (quercetin-7-O-(6-malonyl) glucoside, luteolin-7, 3′-di-O-glucoside, dihydrokaempferol; Figure 4D). There were 304 DCMs identified in the PEG24h vs. CG and 245 DCMs identified in the PEG60h vs. CG when the filter threshold was |log_2_(Foldchange)| ≥ 1 and VIP ≥ 1 (Figures 4E,F). These DCMs were divided into 9 categories: flavonoids, amino acids and their derivatives, phenolic acids, lipids, organic acids, nucleotides and their derivatives, lignin and coumarin, alkaloids and terpenes. There were 12 metabolites over-produced and 292 metabolites under-produced in the comparison of PEG24h vs. CG (Figure 4E and Supplementary Table 5). There were 22 metabolites over-produced and 223 metabolites under-produced in the PEG60h vs. CG (Figure 4F and Supplementary Table 6). In particular, the over-produced DCMs were mainly flavonoids and very long chain fatty acids, indicating that these metabolites could facilitate *P. nutans* to drought stress.

**Figure 5:**
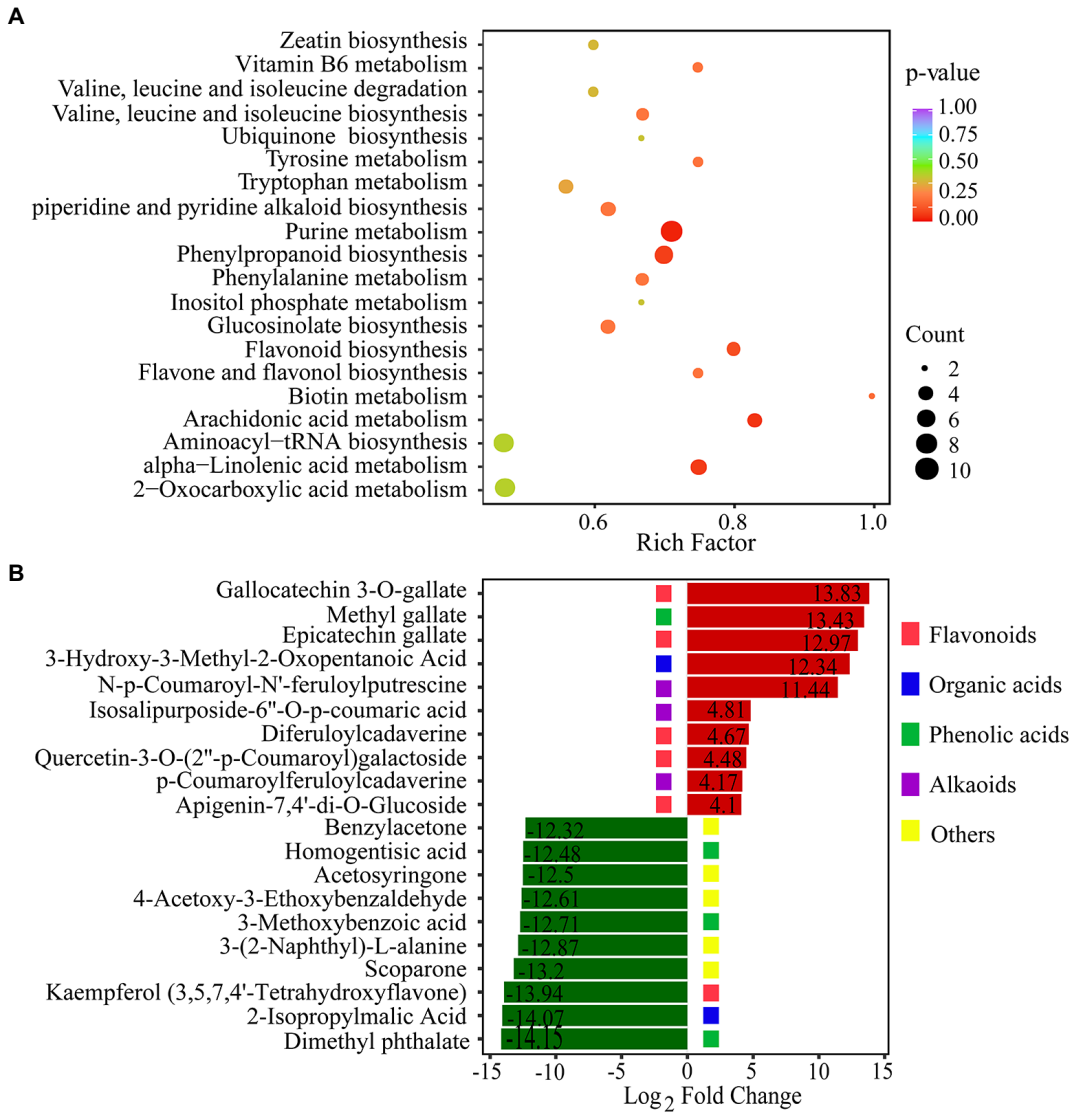
Statistical analysis of differentially changed metabolites (DCMs). **(A)** KEGG enrichment analysis of the DCMs between the PEG60h group vs. CG. Rich factor indicates the ratio of the DCMs amount to the total amount of annotated metabolites in the pathway. **(B)** The fold change of the top 20 significantly changed metabolites between the PEG60h group vs. CG. The numbers into the bars refer to log_2_(Fold change).

To reveal the function of the differential metabolites, KEGG pathway enrichment analysis was performed by rich factor, value of *p*, and the number of enriched genes (Figure 5A). value of *p* was calculated by hypergeometric test. The closer the value of *p* is to 0, the enrichment is more significant. The count of significantly different metabolites enriched in each pathway were also calculated. We used the value of *p* < 0.15 as threshold to select out the key KEGG pathways which have the statistical significance. KEGG pathway analysis revealed that DCMs were mostly enriched in five pathways: purine metabolism, phenylpropanoid biosynthesis, alpha-linolenic acid metabolism, arachidonic acid metabolism, and flavonoid biosynthesis (Figure 5A). In plants, purines are involved in nitrogen metabolism and the formation of metabolic intermediates, including allantoin, which protects plants against stress. There is strong correlation between endogenous allantoin levels and physiological responses under various stresses, such as drought and high salinity. In addition, both allantoin and purines generally enhance tolerance to adverse physiological conditions (Proietti et al., 2021). The phenylpropanoid pathway plays a key role in plant development and defense, as well as in the biosynthesis of flavonoids and phenolic acids (Camas et al., 2012). The top 20 DCMs were shown according to the order of |log_2_(Fold change)| in the comparison of drought stress with CG (Figure 5B). The over-produced DCMs mainly included flavonoids, organic acids, phenolic acids, and alkaloids. Gallocatechin 3-O-gallate was the most significantly DCMs with log_2_(Fold change) of 13.83. Flavonoids and phenolic acids were very abundant in DCMs. Our results showed that there was a substantial accumulation of flavonoids and phenolic acids in *P. nutans* under drought stress.

### Integrated analysis of transcriptome and metabolome under drought stress

**Figure 6:**
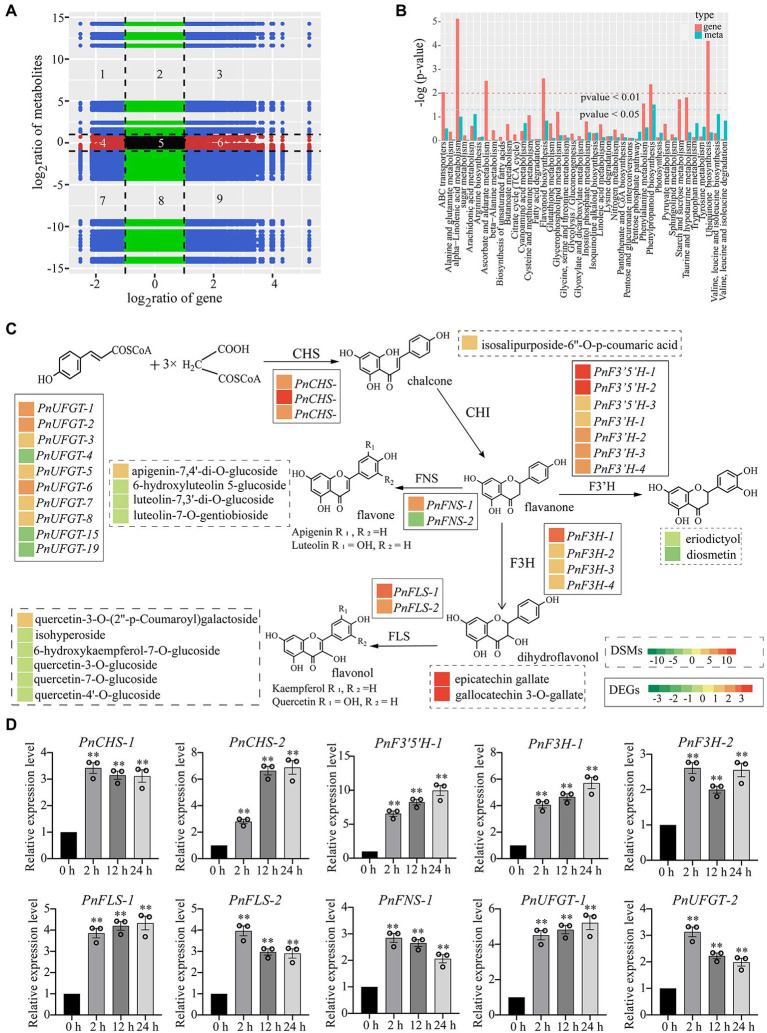
Integrated transcriptome and metabolome analysis highlighted the essential role of flavonoid pathway under drought stress. DCMs in PEG60h vs. CG and DEGs in PEG6000-treatment vs. CG were used for integrated transcriptome and metabolome analyses. **(A)** Correlation between the metabolites and transcript levels of the genes. There were no differential changes in genes and metabolites within the red, green, and black regions. The blue area represents a differential change, which is statistically significant. Pearson correlation coefficients for genes and metabolites were calculated using the cor program in R. **(B)** Integrated analysis of KEGG enrichment pathways for transcriptome and metabolome data. Horizontal ordinate indicates metabolic pathways, red on the ordinate indicates enrichment values for differential genes, and light blue indicates enrichment values for differential metabolites. Vertical ordinate indicates the significance of metabolic pathway enrichment. **(C)** Heatmap analysis of metabolites accumulation and differentially expressed genes in flavonoid pathway. The left color block of each gene and metabolite indicated the log_2_ (Fold change) of this gene and metabolite under drought stress. Genes labeled in solid line box and metabolites showed in dotted box. **(D)** qPCR analysis confirmed that flavonoid synthase genes were upregulated under drought stress. CHS, chalcone synthase; F3′5′H, flavonoid 3′, 5′-hydroxylase; F3H, flavonone 3β-hydroxylase; FNS, flavones synthase; FLS, flavonol synthase; UFGT, UDP-glucose: flavonoid 3-O-glucosyltransferase. The data were calculated from three biological replicates. Vertical bars are means ±SE. Significant difference (**p < 0.01).

To further investigate the relationship between DEGs and DCMs of *P. nutans* under drought treatment, we performed a transcriptome and metabolome co-expression network analysis. We selected DEGs and DCMs with Pearson correlation coefficient greater than 0.8 to draw a histogram. Genes and metabolites with negative correlation in quadrants 1, 2, 4, 6, 8, and 9. Metabolites were overproduced in quadrants 1, 2, and 4, where genes were unaltered or downregulated. However, in quadrants 6, 8, and 9, genes were upregulated, and metabolites were unchanged or under-produced. Only in the 3rd and 7th quadrants, genes and metabolites had positive correlation, and the changes of metabolites might be positively regulated by genes (Figure 6A). We drew a histogram to show the correlative degree of enrichment pathways based on the KEGG enrichment analysis of DEGs and DCMs. DEGs and DCMs enriched in phenylpropanoid biosynthesis pathways were consistent (value of *p* < 0.05), while those in flavonoid biosynthesis and alpha-linolenic acid metabolism were close to being consistent (Figure 6B). These results indicated that flavonoids and fatty acid metabolism might contribute to processes of *P. nutans* fighting against drought stress.

**Figure 7:**
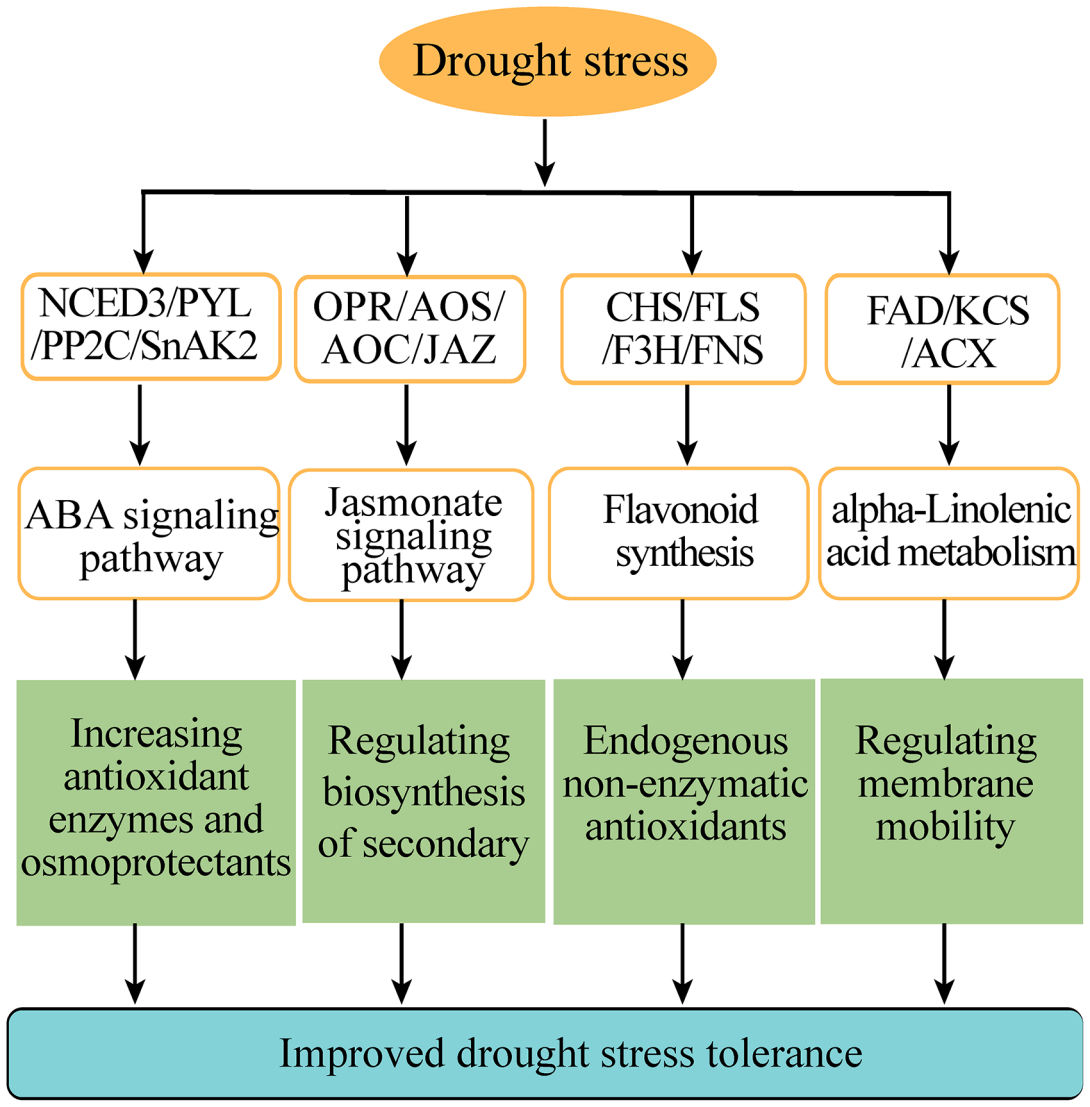
A schematic diagram summarizing the main pathways under drought stress. CHS, chalcone synthase; F3H, flavonone 3β-hydroxylase; FNS, flavones synthase; FLS, flavonol synthase; OPR, 12-oxophytodienoate reductase; AOC, allene oxide cyclase; AOS, allene oxide synthase; JAZ, jasmonate ZIM domain protein; NCED3, 9-cis-epoxycarotenoid dioxygenase; PYL, promoter-pyrabactin resistance; PP2C, type 2C protein phosphatases; SnAK2, SnRK1-activating protein kinase 2; FAD, Fatty acid dehydrogenase; KCS, 3-ketoacyl-CoA synthase; ACX, acyl-coenzyme A oxidase.

Flavonoids are a kind of abundantly secondary metabolites. Many flavonoids act as non-enzymatic antioxidants to combat abiotic stresses. Our metabolome analysis found that chalcone (isosalipurposide-6″-O-p-coumaric acid), flavone (apigenin-7,4′-di-O-glucoside), flavonol (quercetin-3-O-(2″-p-coumaroyl) galactoside) and two flavanols (epicatechin gallate and gallocatechin 3-O-gallate) were significantly accumulated under drought stress (Figure 6C). Meanwhile, the gene expression analysis in transcriptome sequencing revealed the upregulation of several genes encoding the enzymes of flavonoid biosynthesis pathway, including chalcone synthase (CHS), flavonoid 3′, 5′-hydroxylase (F3′5′H), flavonone 3β-hydroxylase (F3H), flavones synthase (FNS), flavonol synthase (FLS), and UDP-glucose: flavonoid 3-O-glucosyltransferase (UFGT; Figure 6C). In particular, qPCR analysis showed that these genes involved in flavonoid biosynthesis were markedly upregulated under drought stress, such as *PnCHS-1*, *PnCHS-2*, *PnF3*′*,5*′*H-1*, *PnF3H-1*, *PnF3H-2*, *PnFLS-1*, *PnFLS-2*, *PnFNS-1*, *PnUFGT-1*, and *PnUFGT-2* (Figure 6D). Taken together, through integrating transcriptome and metabolome data, we speculated that flavonoid and fatty acid biosynthesis, ABA and jasmonate signaling pathways played a synergistic role in enhancing the resilience of *P. nutans* to drought stress (Figure 7).

## Discussion

In the Maritime Antarctica, global warming has led to a rapid warming of the Antarctic Peninsula and is supposed to increase water availability (Torres-Díaz et al., 2016). However, during the last 30 years, the recent analyses have also indicated that the regional temperature has shifted from a warming trend of 0.32°C/decade (1979–1997) to a cooling trend of-0.47°C/decade (1999–2014), especially in the north and northeast of the Antarctic Peninsula and the South Shetland Islands (Turner et al., 2016; Oliva et al., 2017). Meanwhile, the majority of East Antarctica’s regions are experiencing drying climate and colder summer (Robinson et al., 2018). There are a number of urgent ecological and environmental issues related to Arctic and Antarctic mosses in a changing climate (Royles and Griffiths, 2015). Overall, Antarctic mosses only flourish in a short growing season when snow melts and sufficient water is available, but they are frequently stressed by drought and become withered for long periods of time. Until now, few studies have examined the effects of global climate change, especially the changes to regional micro climates, such as drought stress, on the growth, survival, reproduction and molecular responses of Antarctic mosses. Here, to reveal the mechanism of Antarctic moss in response to drought stress, we performed biochemical experiments, as well as a joint transcriptome and metabolome analysis of Antarctic moss *P. nutans* under the treatment of 20% PEG6000 (Figures 1–6). We found that the adaptive responses of Antarctic moss to drought stress were mainly attributed to ABA and jasmonate signaling pathways, flavonoids and fatty acid metabolism, as well as the activities of antioxidant enzymes (Figure 7).

Plant hormone signaling pathway plays a key regulatory role in plant resistance to various biotic and abiotic stresses (Verma et al., 2016). ABA is an important phytohormone responsible for activating drought resistance, which promotes the generation of antioxidants and scavenge reactive oxygen species (Gai et al., 2020). Studies have shown that ABA directly participates in the desiccation tolerance of plants (Khandelwal et al., 2010), and ABA induces expression of genes for LEA-like proteins, enzymes for antioxidant production, and many bryophyte-specific proteins (Takezawa, 2018). Our transcriptome sequencing showed that the DEGs are mainly related to ROS-scavenging and detoxification, flavonoid metabolism pathway, plant hormone signaling pathway, lipids metabolism pathway, transcription factors and signal-related genes (Supplementary Table 3). In Arabidopsis, there is strong evidence that the ABA receptors PYL promotes resistance to extreme drought stress (Zhao et al., 2016). Our transcriptome analysis also revealed that the ABA receptor PYL was upregulated in *P. nutans* under drought stress, indicating that the ABA signaling pathway is involved in drought resistance. Furthermore, our qPCR analysis showed that the ABA synthase gene *NCED3*, the ABA receptor *PYL*, *PP2C*, and *SnAK2* were all upregulated (Figure 3A). Interestingly, the moss *P. patens* contains JA precursor (OPDA) but not JA or its amino acid conjugates, which indicates that OPDA regulates a signaling pathway distinct from JA (Dave and Graham, 2012). Additionally, *Arabidopsis thaliana* potently resists the damage of drought stress by the contribution of OPDA, which was produced by a branching process of allene oxide synthase (AOS; Savchenko and Dehesh, 2014). 12-Oxophytodienoate reductases (OPRs) are homologues of old yellow enzymes, which represent a class of FMN-dependent oxidoreductases. Wheat *OPR* has been shown to improve the plant tolerance to high salinity by increasing *MYC2* expression, which in turn activates an ABA-dependent signaling pathway (Dong et al., 2013). We found that jasmonate pathway genes were upregulated, including *OPR*, *AOS*, *AOC*, *and JAZ* through transcriptome sequencing and qPCR analysis (Figure 3B). Jasmonates are crucial signals that regulate the biosynthesis of secondary metabolites, especially in modulating flavonoid biosynthesis. In addition, jasmonates are relatively documented in terms of their roles in regulating biotic and abiotic stress responses, development and metabolite synthesis in basal land plants like bryophytes.

We performed a widely targeted metabolome analysis using an UPLC-MS/MS platform. Among the DCMs, lipids accounted for 32.5% (Supplementary Table 6). It is worth noting that lipids contain ultra-long-chain fatty acids (VLCFAs). VLCFAs are structurally and functionally diverse molecules due to their carbon chain length, degree of unsaturation, and the polarity of the head structure, and play key roles in a variety of physiological processes such as transmembrane transport, energy storage, and resistance to stress (Zhukov and Shumskaya, 2020). Combining the metabolome and transcriptome analysis, we inferred that the upregulated 3-ketoacyl-CoA synthase (*KCS*) might promote the synthesis of VLCFAs (Supplementary Table 3). The KCS-catalyzed condensation reaction is a key rate-limiting step in the elongation of long-chain fatty acids, which has the substrate-and product-specificity and determines the carbon chain length and content of VLCFAs (De Bigault Du Granrut and Cacas, 2016). Over-expression of *KCS* can enhance resistance to drought stress by changing the composition of VLCFAs in cuticular wax (Lian et al., 2021). Currently, only two KCS genes (*MpFAE2* and *MpFAE3*) of *Marchantia polymorpha* are known to synthesize beta-ketoacyl-CoA synthase (Kajikawa et al., 2003a,b). In the present study, we speculated that KCS might play an important role in drought resistance in *P. nutans*. However, the regulatory mechanism of KCS regulating drought stress in Antarctic bryophytes still remains unclear.

Our metabolome analysis revealed that the metabolites with significant changes were involved in phenylpropanoid biosynthesis, flavonoid biosynthesis and alpha-linolenic acid metabolism (Figure 4A). We found that most of the top 20 DCMs were related to flavonoids and lipid metabolism (Figure 4B). Flavonoids are a class of secondary metabolites found in plant and fungus (Sheng et al., 2020). Flavonoid accumulation often occurs in plants under abiotic stresses, including the plants adaptation to the environment and overcoming adverse conditions (Singh et al., 2017). In bryophytes, the flavonoid biosynthesis pathway and its regulatory mechanism are not as well characterized as in angiosperms (Davies et al., 2020). CHS is the first enzyme involved in plant flavonoids biosynthesis, and it occurs in all land plants (Liou et al., 2018). In addition, previous studies confirmed that the mosses F3′Hs and F3′5′Hs are highly conserved and have evolved independently from monocots, dicots and ferns (Liu et al., 2014). In this study, three CHS genes and six F3′5′H genes were identified from the transcriptome of the Antarctic moss *P. nutans* (Supplementary Table 3). Furthermore, qPCR results confirmed the upregulation of *CHS, F3*′*5*′*H, F3H, FLS, FNS* and *UFGT* in *P. nutans* under drought stress (Figure 6D). Through metabolome analysis, we found that flavonoids were the main differential metabolites, with three over-produced metabolites (i.e., Gallocatechin-3-O-gallate, Methyl gallate and Epicatechin gallate). These three metabolites were classified as flavonoids. Methyl gallate has the ability to block the production of ROS and inhibit the production of inflammation in the kidneys of the human body (Liu et al., 2021a). Gallic acid derivatives could also be DNA replication inhibitors as some of the green tea extracts (López-Lázaro et al., 2011). Gallocatechin-3-O-gallate and Epicatechin gallate would be suppressing the production of toxic reactive metabolites (Yasuda et al., 2021). These flavonoids might play a synergistic role with the upregulation of antioxidant enzymes and detoxification proteins in ROS-scavenging (Supplementary Table 3). Furthermore, histogram plot showing the correlative degree of DEGs and DCMs enrichment pathways demonstrated that several stress-related pathways were highly enriched, including phenylpropanoid biosynthesis, flavonoid biosynthesis, and alpha-Linolenic acid metabolism (Figure 6B). In conclusion, our results suggested that flavonoid biosynthesis, fatty acid biosynthesis, ABA and jasmonate signaling were the main protecting mechanism of Antarctic moss in response to drought stress. These results will help us to further assess the profound impacts of coastal climate change on these Antarctic basal land plants.

## Data availability statement

The original data presented in the study are publicly available. The whole genome sequence data were available in the National Genomics Data Center (NGDC, https://ngdc.cncb.ac.cn) under the BioProject number PRJCA008231. The genome assembly and annotation data were deposited in Genome Warehouse (GWH) under the accession number GWHBHNB00000000. The transcriptome sequencing data of *Pohlia nutans* were also deposited in the Genome Sequence Archive (GSA, https://ngdc.cncb.ac.cn/gsa/) under the BioProject number PRJCA008962 and the sequence accession number CRA006553.

## Author contributions

SL and CL conceived the original research and designed the experiments. SF conducted the biochemical features and quantitative real-time RT-PCR experiments, analyzed the data and wrote the draft manuscript. TL finished the transcriptome sequencing and analysis. PZ performed the metabolite extraction and LC-MS/MS analysis. BC collected the plant samples, moss culture and conducted drought stress treatment. SL revised the manuscript. All authors contributed to the article and approved the submitted version.

## Funding

This work was supported by the National Natural Science Foundation of China (41976225) and Central Public-Interest Scientific Institution Basal Research Foundation of China (GY2019Q05).

## Conflict of interest

The authors declare that the research was conducted in the absence of any commercial or financial relationships that could be construed as a potential conflict of interest.

## Publisher’s note

All claims expressed in this article are solely those of the authors and do not necessarily represent those of their affiliated organizations, or those of the publisher, the editors and the reviewers. Any product that may be evaluated in this article, or claim that may be made by its manufacturer, is not guaranteed or endorsed by the publisher.
