# Supplementary material for: Integrated transcriptome and metabolome analyses reveal the adaptation of Antarctic moss Pohlia nutans to drought stress
Source: Front Plant Sci. 2022 Aug 11;13:924162. doi: 10.3389/fpls.2022.924162 (PMC9403716; doi:10.3389/fpls.2022.924162)
Supplement: Supplementary file 1 [file Table_1.DOCX]

**Supplementary Table 1** Primers used in quantitative RT-PCR analysis.

| Gene ID | Gene symbol | Primer name | Primer sequence (5’-3’) | Melting temperature (°C) | Product length (bp) |
| --- | --- | --- | --- | --- | --- |
| Poh0049620.1 | *PnPOD-1* | PnPOD-1F | CCGTGAGAACAACGCCAAGGT | 59.3 | 200 |
|  |  | PnPOD-1R | TGAAAGCAAACTCCGCCACCAG | 59.9 |  |
| Poh0231250.1 | *PnPOD-2* | PnPOD-2F | ATGGCTGCTCTGCTTGTTGTGA | 59.1 | 179 |
|  |  | PnPOD-2R | TCGTGGAAATGGAGGCGGACTA | 59.5 |  |
| Poh0217290.1 | *PnPrx-1* | PnPrx-1F | GTACTTCGACACGGACGGCAAT | 59.3 | 196 |
|  |  | PnPrx-1R | ATCGTTCACGGAGACGCAAGC | 59.5 |  |
| Poh0041980.1 | *PnGSH-PX1* | PnGSH-PX-1F | GCGGTTCAAGGCGGAGTATCCT | 59.1 | 195 |
|  |  | PnGSH-PX-1R | TTGGAAGGAGCAGTGGTCGGT | 57.1 |  |
| Poh0002580.1 | *PnDTX-1* | PnDTX-1F | GGTGGTGCCAGTGGAGTTGAA | 58.6 | 167 |
|  |  | PnDTX-1R | TGAAAGCCTCTCCCAAGCCAAG | 58.8 |  |
| Poh0270550.1 | *PnCHS-1* | PnCHS-1F | ACCAACGGACCAATTCGCTTCA | 59.1 | 154 |
|  |  | PnCHS-1R | GCCTTCCATGCTGAGTCCTGTC | 59.3 |  |
| Poh0222480.1 | *PnCHS-2* | PnCHS-2F | GCCGGATCAGACGAGCAAGTT | 58.9 | 191 |
|  |  | PnCHS-2R | TCGCTGGTGTTGGTGATGTTGA | 58.8 |  |
| Poh0016430.1 | *PnF3′5′H-1* | PnF3′5′H-1F | GCCGCTCGCCAACATCAACAT | 60.4 | 157 |
|  |  | PnF3′5′H-1R | CGCAACAGCAAGGAGAGGACTC | 59.6 |  |
| Poh0311510.1 | *PnF3H-1* | PnF3H-1F | ACTTCGCCTCGTATGCCGTGTT | 60.9 | 163 |
|  |  | PnF3H-1R | TTCCTCCGTTCCTCCCGTCCTT | 61.1 |  |
| Poh0117410.1 | *PnF3H-2* | PnF3H-2F | CCCACGGCGGTCATTGAGAAA | 59.1 | 147 |
|  |  | PnF3H-2R | GCCAGCGTAGAAGAAGCCACTC | 59.6 |  |
| Poh0176910.1 | *PnFLS-1* | PnFLS-1F | ATGGCACCAGAGGCACAGAGT | 59.6 | 163 |
|  |  | PnFLS-1R | TCGCCACCTTCAGCCTTGTCT | 59.8 |  |
| Poh0038150.1 | *PnFLS-2* | PnFLS-2F | AGGAGAAGGTGGCGAAGGAGAT | 58.9 | 195 |
|  |  | PnFLS-2R | TGTAGCCGAAGGAGTTGTTGGA | 57.7 |  |
| Poh0283250.1 | *PnFNS-1* | PnFNS-1F | CCAGGCGATTACCAACGGCATA | 59.1 | 189 |
|  |  | PnFNS-1R | TCTCGGTAGGCGTTGACAGTGA | 59.4 |  |
| Poh0253890.1 | *PnUFGT-1* | PnUFGT-1F | CGACCATCACAATCCGCCTCTG | 59.7 | 193 |
|  |  | PnUFGT-1R | AGCACTTCCAACTCCAGCCATC | 58.9 |  |
| Poh0077500.1 | *PnUFGT-2* | PnUFGT-2F | TGTCGGTGGCTGGAGTTGGAA | 59.8 | 150 |
|  |  | PnUFGT-2R | CCGCAGCATACTCACGCATCT | 59.1 |  |
| Poh0219930.1 | *PnOPR-2* | PnOPR-2F | ACCCTGGCTACCGACATGATGA | 59.2 | 140 |
|  |  | PnOPR-2R | ACTTGCTCGCTCGTGTAGATCC | 58.6 |  |
| Poh0027780.1 | *PnAOC-1* | PnAOC-1F | CGACAGGCATCTGACGAAGCA | 58.9 | 198 |
|  |  | PnAOC-1R | GGCGTGATGTTGTGGAGGTGAA | 59.4 |  |
| Poh0112930.1 | *PnAOS-1* | PnAOS-1F | AACAGTGGTGCTTCGACTTGGT | 58.7 | 145 |
|  |  | PnAOS-1R | AACTGCTGAACCTCCGCTGAC | 58.8 |  |
| Poh0024230.1 | *PnJAZ-1* | PnJAZ-1F | CTGCTCCTGACCGTGACTCGTA | 59.8 | 148 |
|  |  | PnJAZ-1R | TGGTTGGAAGGGTTGTGCTCTG | 59 |  |
| Poh0281560.1 | *PnJAZ-2* | PnJAZ-2F | TCTCCAACATTCGACCACCACC | 58.6 | 130 |
|  |  | PnJAZ-2R | GCTTCGCCTTCCTCACTCACA | 58.5 |  |
| Poh0286330.1 | *PnNCED3-1* | PnNCED-1F | CCAAGGAAGACGACGGGTATGT | 58.1 | 190 |
|  |  | PnNCED-1R | GATGACCTCTGGCTGGCAAGTT | 58.9 |  |
| Poh0347020.1 | *PnPYL-2* | PnPYL-2F | CGAGGAGGAGCACATCATCAGT | 57.9 | 198 |
|  |  | PnPYL-2R | CGCACAACGGTATCGACGAACA | 59.9 |  |
| Poh0326080.1 | *PnPYL-3* | PnPYL-3F | GACGAGGAGGAGCACATCATCA | 57.9 | 200 |
|  |  | PnPYL-3R | CGCACAACGGTATCGACGAACA | 59.9 |  |
| Poh0354190.1 | *PnPP2C-1* | PnPP2C-1F | AGACGGACAAGGAATGGCAGAA | 58 | 122 |
|  |  | PnPP2C-1R | GGCATCTAGCACACACCGAGAA | 58.7 |  |
| Poh0034220.1 | *PnSnAK2-1* | PnSnAK2-1F | TTGTGGTCCTCCAGGTGGCATC | 60.5 | 147 |
|  |  | PnSnAK2-1R | ACAGTCCCATCTCCGCTTACGA | 59.2 |  |
| Poh0061050.1 | *PnERF-1* | PnERF-1F | CGCCGTTGAAGAAGCCATTGTC | 59.1 | 133 |
|  |  | PnERF-1R | CGAATCTCGCTCACCCATTTCC | 57.8 |  |
| Poh0220890.1 | *PnERF-2* | PnERF-2F | AGTCGTCGTCGTCGGTGAATC | 58.4 | 135 |
|  |  | PnERF-2R | GGAACGAGCCCAACCAAATGC | 58.6 |  |
| Poh0057850.1 | *PnERF-3* | PnERF-3F | CAGCGGAGAAGGAGAGCGAAAC | 59.6 | 200 |
|  |  | PnERF-3R | GAAGTTGAGCGTGGCGTTGGA | 59.7 |  |
| Poh0333760.1 | *PnDREB-1* | PnDREB-1F | CGATGACGAGAGCGACGAGTT | 58.5 | 127 |
|  |  | PnDREB-1R | AGGTTGCGAGGATGAAGACGAA | 58.1 |  |
| Poh0133760.1 | *PnDREB-2* | PnDREB-2F | CGGCACGAGGATGAGGAAGAAG | 59.1 | 160 |
|  |  | PnDREB-2R | CATTCAAGACGCTGTCGAGGCT | 59.3 |  |
